# Fetor from a Dental Stand-Point

**Published:** 1900-08

**Authors:** B. Holly Smith

**Affiliations:** Baltimore, Md.


### FETOR FROM A DENTAL STAND-POINT.[1]

BY B. HOLLY SMITH, BALTIMORE, MD.

MR. PRESIDENT AND MEMBERS OF THE ACADEMY OF STOMATOLOGY,—In the world of commerce, of sale and exchange, men buy and trade for what they want upon such terms as they can make. As a rule, the man who sells does not guarantee a profit to the man who buys when he comes to sell again. Yet even in this world the permanence of business relations between buyer and seller depends upon the ability of the seller to furnish an article upon the sale of which the buyer may make a living profit. Thus a more or less hard-and-fast system of interdependence exists, the wholesaler advising and conferring with the retailer as to the kind and quality of goods which he thinks best suited to the retail trade. In like manner the clerk in the retail store will advise the customer as to the quantity, quality, or color of goods required.

In the domain of professional life this dependence of one upon another, which is but faintly foreshadowed in the business world, becomes a fixed and definite principle. The patient, having selected his adviser, as a rule no longer seeks to protect his own interests, except by following the instructions of that adviser. The adviser must be honest, because he is trusted; his attentions must be helpful, because the subject is helpless. The doctor assumes parental authority over his patient, thus becoming a teacher as well as a practitioner; and just in proportion as those practising our specialty appreciate and live up to this high calling will we be regarded as discharging our full obligation to our patients, and taking our place beside other specialists of the great healing art. This contention—trite and old, if you please, yet bearing the spark of vital-

---

[1] Read before the Academy of Stomatology, March 27, 1900.

ity in that it seeks the perpetuation of the life and health of our calling—is my excuse for reciting in your hearing some clinical experiences which, let us hope, will help to establish the thought that the dentist is responsible not alone for the condition of the teeth, but for oral sanitation generally.

The olfactory sense may be regarded as one of the so-called *artistic* senses, and is capable of training and education. People of refinement in all ages have striven to avoid offending it, and not infrequently efforts are made to contribute to the pleasure of the individual through this sense. The delicate perfume of the flower appeals as much to the artistic sense through the olfactories as the coloring of the gracefully moulded petals do through the optics. So well established is this fact that women of refinement vie with each other in the effort to secure a slight though characteristic perfume for their garments, hair, and person. Some have gone so far as to endeavor to associate this perfume with their identity. It is said that whenever Mr. Browning—whose love passes everywhere for ideal—came into the presence of Mrs. Browning he was conscious of the faint odor of violets. And it is intimated that this was a psychic phenomenon produced in the olfactory sense by the extreme mental ecstasy caused by the presence of the adored.

Persons of refinement and culture, of beauty, grace, and good taste, should spread around them everywhere pleasurable impressions. How often do we see these charms negated, and social intercourse become burdensome because of a fetid breath.

To me there is absolutely nothing more beautiful than the human mouth in a state of perfect health, beaded above and below with the most ornamental and precious set of gems, whose beauty is partly concealed by the gracefully curved lips, the pink coloring of which serves to heighten their lustre, the cheeks, the tongue, the palate far back into the pharynx rosy with the tint of health and glistened by the limpid libations of a generous though not too active salivary apparatus, while out and over these comes the expiration of a breath like that of a May morning, having the sweet, fresh, inimitable odor of health.

The treatment of fetor as the subject of a paper may not find favor in the eyes of those who recognize it only as a symptom of some disease and never as a special condition, the amelioration of which requires special care and treatment aside from that prescribed for the many conditions which have it for a symptom. To these let me say that I have selected the subject partly because of the existence of fetor as a special factor in a number of cases of no little interest to me, partly because I think the dentist above any other specialist contributes to the amelioration of this condition, and more than all because I want to establish the contention that the patient must look to the dentist and make him responsible for such relief.

Even though the fetor has nothing to do with the mouth or the area usually included in the dentist's field of work, in the close contact which this specialist sustains to his patient he must be able to recognize its existence and should suggest the propriety of seeking treatment for conditions which the patient seems disposed to neglect. It must be conceded that while other specialists often direct their attentions to the amelioration of conditions giving rise to fetor, the dentist is the specialist most likely to detect its existence; and it appears to me to be his plain duty to determine its cause; if he can do so, to remove it, if not, he should direct his patient to the specialist best fitted to do so.

Fetor may be said to indicate always a departure from the normal, though its presence is not in every case coexistent with illness of a serious nature. Many persons seem oblivious to it, and medical attention is not always sought even when it is recognized. A mistaken sense of refinement often causes members of a family to conceal their knowledge of its existence from the sufferer, and when the patient becomes aware of it he is ignorant of its cause.

Among the conditions in which fetor plays an important part in determining the presence and progress of disease we may mention bromine, iodine, mercurial, lead, and phosphorus poisonings; caries of the teeth; loose or badly constructed bridges and crownwork; unsanitary plates and appliances; alveolar abscess, pyorrhœa alveolaris; various inflammations, specific and non-specific, of the mouth and appendages; fevers, constipation, diarrhœa; in fact, any departure from normal health and tone.

The mouth is lined by pavement epithelium, which is embryologically of the same origin as the epidermis of the skin, and therefore does not belong to the mucous membrane in the same sense as the lining of the stomach, bronchi, etc. There are numerous mucous glands in the mouth which open on the inner surface of cheeks, lips, and on the surface or beneath the tongue. The excretion of these is added to that of the salivary glands to make the complete fluid of the mouth. Many substances, both mineral and alkaloidal, which are absorbed into the circulation are eliminated by these glands as well as the salivary glands, and in being secreted have their influence on the mucous membrane of the mouth. It is a well-known fact that mercury, after it has gotten into the circulation either through absorption by the skin from inunction or by hypodermic injection, is excreted by the mouth, and may give rise to a stomatitis just as severe as if an irritant were applied to the mucous membrane of the mouth. This is in accordance with the well-known law that substances which are secreted or excreted by mucous membranes act on those mucous membranes.

We notice that these drugs generally cause death to the superficial layers of epithelia, and of course dead epithelium is at once attacked by the saprophytic bacteria of the mouth, notably the staphylococcus pyogenes fœtida.

Miller has shown that these dead epithelia and thickened mucous secretion scraped from a furred tongue show the presence of many rapidly developing organisms. It is only, however, when these organisms act on the dead epithelia and secretions in some little pocket, such as those around the teeth, in the valleculæ of the circumvallate papillæ, or in the crypts in and around the lingual and pharyngeal tonsil, where the air has not free access, that odor occurs.

We find much the same phenomena occurring after the administration of some of the vegetable alkaloids, which are eliminated by the mouth. Atropine checks the secretions of the mouth, and, if pushed, is followed by furred tongue, etc. Other alkaloids also are excreted by the glands of the mouth, and in passing out act on the mucous membrane.

Now, I have no doubt that a similar explanation may be given for the furred tongue and bad breath occurring in many constitutional diseases, such as the fevers, etc. Modern investigation has shown that in the course of these diseases there are developed substances (generally the product of pathogenic organisms) called toxines, which are nitrogen compounds resembling in chemical construction and often in action many of the vegetable alkaloids; the greatest difference is that they are derived from animal instead of vegetable tissues. It seems to me much more logical to refer the furred tongue to the superficial necrosing effect of these toxines during their secretion by the mucous membrane of the mouth than to say it is in sympathy with the other organs of the body, stomach, etc. I do not believe it is a spreading of the condition of the stomach up the long œsophagus to the mouth so much as a simultaneous affecting of both by toxines secreted from the blood by the glands and epithelia of both.

Again, the furred tongue of constipation should be explained in a similar way. It is only one of the many evidences which the body shows of fecal poisoning, making its appearance here much sooner than in other portions of the body. It is not necessary to go into the subject of fecal poisoning to discuss what substances are absorbed from decomposing fæces blocked up in the bowels Chemistry of the animal products, or physiological chemistry, as it has been termed, which has been developed so much lately since Vaughn extracted his tyrotoxicon from milk and cheese, has shown us that many of the phenomena of disease are to be traced to these products of animal matter,—namely, toxines in living bodies and ptomaines in dead.

Normal fæces contain, besides the residuum of digestion, substances which are excreted, or not intended for use in the economy, but are rather products which are deleterious; such as those in the bile, etc. Now, when the fæces do not pass out in a reasonable time, we have in addition the products of putrefaction, which are even more deleterious. These are absorbed into the blood and carried all over the body, and the mucous membrane of the mouth in eliminating them is affected thereby.

Now, what is the cause of the odor imparted to the breath? These cases of furred tongue in fevers, fecal poisoning, etc. We cannot suppose that such substances may be excreted by the mucous membrane of the mouth without causing a mild degree of stomatitis. Many of the epithelial cells become opaque and lose their vitality, the secretions of the glands are modified, and these conditions afford dead material, which the saprophytes, always present in the mouth, develop. Whenever there is a space where these grow and the air does not get free access, as in the valleculæ at the base of the tongue, the pockets between the gums and teeth, or even where the dead epithelia is so thick as to exclude the air from the lower layers, gases may develop and impart an odor to the breath.

Not infrequently the adenoid tissue known as the lingual tonsil is the seat of cheesy deposit or abscess, which gives rise to fetor.

The thick, ropy secretions which are often stagnant behind the folds of the velum palati, between the tonsils, or about the uvula, are often fetid. In the act of sneezing these are brought in the mouth, when the fetor is readily discovered. When the teeth are being cleaned with the brush, if the bristles be pressed for back on the tongue and palate, these secretions will be dislodged by gagging. Patients should be instructed to cleanse the mouth and appendages.

The following are some of the cases, the report of which it is thought might assist in making the application of the attempted teaching of this paper.

Miss M., cashier in a toy-store, had been in bad health for several months; trouble began with what was pronounced la grippe by the homœopathic physician under whose care she remained. After much suffering and intermittent employment, the patient sought dental services for the relief of an aching tooth. On account of pronounced fetor a careful examination was made, suppurating antrum discovered, successfully treated, and patient restored to health.

A number of antrum cases could be mentioned where fetor was the pronounced symptom and where it assisted the diagnosis, but this case is cited because of the fact that but for the fetor the antral suppuration would not have been discovered and prompt relief afforded.

Miss B., who was under my care, was a very handsome and vivacious young woman. I noticed that in spite of the fact that her teeth were in good condition and the mucous membrane had a healthy appearance, her breath had a fetid odor. I called her attention to it so that we might co-operate in finding the cause. A superficial examination of the nasal cavities was made without discovering any cause for the fetor; the throat seemed normal. She was habitually constipated, so means were suggested for the relief of this condition, and she was requested to call in about two weeks. In the mean time I had a visit from her mother requesting that everything be done for the young lady which was possible. Upon her return, she reported improvement in habit, but there was the same close fetid taint to the breath. In a more careful examination of the throat and tongue, I noticed that the circumvallate papillæ on the dorsum lingualis were very prominent, and a spoon-shaped spatula scraped over this part of the tongue collected mucus of a most fetid character. With a delicate bistoury I cut through one of these papillæ, and found by the side of it an accumulated mass of very fetid matter. Upon consultation with a friend, a throat and nose specialist, we decided to obliterate these fungiform papillæ with electric cautery, which was done with absolute relief of the condition.

A case in the practice of Dr. Cyrus M. Gingrich, of Baltimore: Miss P., a young woman of more than ordinary attractions, cultivated and refined. Members of the family complained that her breath was fetid; the mouth was in apparently healthy condition, teeth not responsible, tongue slightly furred, and mucus scraped from it offensive. A tongue scraper was advised, and the tongue cleansed with a weak solution of carbolic acid; after treatment a radical cure was effected.

A case in the practice of the late Professor Winder: Mrs. L., a lady of position and wealth, had been under the care of a throat specialist for six months, being treated for catarrh of the Schneiderian membrane, with little improvement. She was obliged on account of extreme fetor to forego social pleasures; conscious of the infirmity, she apologized profusely for having to subject her dentist and friend to the ordeal of attending to her teeth. A careful examination of her mouth revealed a pulpless bicuspid which was slightly tender to percussion. Its extraction was advised and consented to; a diseased antral cavity was found and restored to health; the patient, from being a recluse and invalid, once more resumed her position of prominence in her family and social circle.

Mrs. C., a woman of not very robust constitution, has been under my care for several years. A not very pronounced fetor was discovered at first sitting, and referred to the presence of an unusually large proportion of pulpless teeth; the four incisors and four bicuspids in the upper jaw were in this condition. A small fistula, open and empty, was discoverable near the frænum of the upper lip; looked to be an opening from the right central incisor. No examination of the nasal cavities was made at first sitting, as the patient was regarded as a transient, having only applied for specific operations upon molar teeth, two of which, in the upper jaw, were pulpless; there were also several teeth in this condition in the lower jaw. At a subsequent visit it was learned that the subject was under treatment of a specialist for nasal catarrh. A year after, this patient reported no improvement of special condition, general health not good; invalidism threatened, patient discouraged and gloomy; applied to have filling renewed in right superior second molar; tooth found to be pulpless, but pulp-cavity not exposed. Marked fetor was discovered upon opening; then it was found for the first time that some fifteen years previous a great deal of dentistry was done under the influence of a new obtunding agent; and whereas all dentistry previous to this was performed with a great deal of pain, this was done after treatment and without any pain at all. The arsenical "negro in the wood-pile" being suspected, all pulpless teeth were opened, and most of them found to be in fetid condition. A fistula was discovered in the floor of the left nostril. After a month's treatment fetor disappeared, and after a lapse of three years no more catarrhal symptoms have appeared, the patient is restored to health; the upper teeth, which were diseased and unsightly, have been bleached, and the woman is as sweet and handsome as she was in her fifteen-year-old form.

The fetor attending fractures is in proportion generally to the comminution, where specula or fragments are broken away; these frequently necrose, and quite a characteristic odor is developed. Added to this a fetor from accumulated *débris* about the splint is to be combated.

A fetid odor is often imparted to the breath from the socket where a tooth has been extracted recently. A partially erupted wisdom-tooth about the crown of which the gum is swollen gives a like odor.

I had a patient call for examination whose breath was most fetid. He apologized for it on the ground that his stomach had been out of order. I soon discovered a loose bridge to be the cause of the fetor, but being interested in his diagnosis, I asked him how long he had been suffering. He said that about a month before he had noticed the bad odor when he arose in the morning. He consulted his doctor, who, upon an examination of his tongue, said he would require some purgative medicine, which he took with abundant effect but very little benefit. Since that he had been undergoing a regular course of medicine for indigestion, with not much improvement. He was restored completely by a resetting of the bridge.

On several occasions I have had occasion to warn patients of mercurial salivation, the presence of which I was led to determine by the peculiar fetor associated with this condition.

Your essayist has found permanganate of potash a rock of defence against fetor in all conditions, using it in weak solution, say one grain to the ounce, often having it decolorized by injecting it into suppurating cavities.

